# Study on Enhancing the Thermoelectric Stability of the β-Cu_2_Se Phase by Mn Doping

**DOI:** 10.3390/ma16145204

**Published:** 2023-07-24

**Authors:** Jian Tie, Guiying Xu, Yawei Li, Xian Fan, Quanxin Yang, Bohang Nan

**Affiliations:** 1 Beijing Municipal Key Lab of Advanced Energy Materials and Technology, School of Materials Science and Engineering, University of Science and Technology Beijing, Beijing 100083, China; qhutiejian@163.com (J.T.);; 2 College of Physics and Electronic Information Engineering, Qinghai Normal University, Xining 810016, China; 3 The State Key Laboratory of Refractories and Metallurgy, Wuhan University of Science and Technology, Wuhan 430081, China

**Keywords:** Cu_2_Se, Mn doping, thermoelectric, superionic conductor

## Abstract

Cu_2_Se is a promising thermoelectric (TE) material due to its low cost, Earth abundance, and high thermoelectric properties. However, the biggest problem of Cu_2_Se is its unstable chemical properties. In particular, under the action of an electric field or gradient temperature field, the chemical potential of copper ions inside the material increases. When the external field is strong enough, the chemical potential of copper ions at the negative end of the material reaches the chemical potential of elemental copper. Under these conditions, copper ions must precipitate out, causing Cu_2_Se to be unstable, and making it unsuitable for use in applications. In this study, we prepared Cu_2−x_Mn_x_Se (x = 0, 0.02, 0.04 and 0.06) series bulk materials by vacuum melting–annealing and sintered by spark plasma sintering (SPS). We investigated the effects of Mn doping on the composition, microstructure, band structure, scattering mechanism, thermoelectric properties, and stability of Cu_2_Se. The results show that Mn doping can adjust the carrier concentration, promote the stabilization of the β-phase structure and improve the electrical properties of Cu_2_Se. When x = 0.06, the highest power factor (*PF*) value of Cu_1.94_Mn_0.06_Se at 873 K was 1.62 mW m^−1^ K^−2^. The results of carrier scattering mechanism analysis based on the conductivity ratio method show that the sample doped with Mn and pure Cu_2_Se had the characteristics of ionization impurity scattering, and the scattering factor was 3/2. However, the deterioration in thermal conductivity was large, and a superior *zT* value needs to be obtained. The cyclic test results of high-temperature thermoelectric properties show that Mn doping can hinder Cu^+^ migration and improve its thermoelectric stability, which preliminarily verifies the feasibility of using the stable zirconia mechanism to improve the thermoelectric stability of Cu_2_Se.

## 1. Introduction

TE technology, which enables direct energy conversion between heat and electricity and can directly convert waste heat generated by industrial plants or automobiles into useful electricity, is one of the most promising technologies for improving energy efficiency and has attracted widespread interest for decades [1]. TE materials for civilian use should have high thermoelectric properties and good stability, and have characteristics that lead to financial and environmental benefits. The properties of thermoelectric materials are evaluated using a dimensionless quality factor value *zT* = *σS*^2^ *T*/(*κ_e_* + *κ_l_*), where *σ* is electrical conductivity; *S* is the Seebeck coefficient; *T* is the absolute temperature; and *κ* is the total thermal conductivity, consisting of electron thermal conductivity (*κ_e_*) and lattice thermal conductivity (*κ_l_*) [2]. Therefore, an excellent thermoelectric material must have high *σ*, and *S*, and low *κ*. Many methods have been used to optimize these thermoelectric parameters, such as changing the band structure [3], introducing resonant energy levels [4], and de-dimensionalization, ionization, or alloying to induce scattering [5]. One of the most influential and easiest ways to do this is by doping elements in the alloy.

In recent years, Cu_2_Se with a “phonon liquid–electron crystal” (PLEC) [6] structure has attracted much attention as a promising P-type thermoelectric material, not only because of its excellent thermal (ultra-low thermal conductivity) and electrical (high conductivity) transport properties, but also because of its unique properties such as environmental friendliness, low cost, low toxicity, rich ores, and Earth abundance, high *PF* value, and high *zT* value [7]. Cu_2_Se exhibits P-type conductivity with high carrier concentration at relatively low temperatures, while ionic conductivity becomes obvious at temperatures above 400 K due to the transition from the α-Cu_2_Se to β-Cu_2_Se high-temperature phase [8]. β-Cu_2_Se is a valuable thermoelectric material in which liquid Cu ions are dynamically disordered within a face-centered cubic framework of Se [9]. The excellent thermoelectric properties of Cu_2_Se mainly depend on the anion having a simple lattice structure sublattice. At the same time, the cation is highly chaotic with liquid-like mobility and specific liquid superionic properties, which have an additional scattering effect on the lattice phonons, further hindering heat conduction or changing phonon transfer so that the mean free path of phonons is close to the minimum wavelength of glass. Thus, the material’s thermal conductivity is extremely low [10]. As a TE material, the biggest problem of Cu_2_Se is its unstable chemical properties. That is, the chemical potential of copper ions inside the material increases under the action of an electric field or gradient temperature field. When the external field is strong enough, the chemical potential of copper ions at the negative end of the material reaches the chemical potential of copper elements, and copper ions are precipitated as simple elements [11], making practical application impossible. This instability can be partially mitigated by adding impurities that cause lattice distortion and impede the migration of Cu ions in β-Cu_2_Se. However, current chemical doping is mainly achieved by the addition of transition metals (Fe [12], Mn [13], Ni [14], Ag [15]) or alkaline elements (Li [16], Na [17], K [18], Mg [19]) instead of Cu, or halogens or sulfur elements (I [20], Cl [21], S [22], Te [23]) instead of Se regulate the thermoelectric properties of Cu_2_Se to optimize the thermoelectric properties of Cu_2_Se. This paper proposes three basic principles for stabilizing cation (Cu^+^) for Cu_2_Se series materials with “phonon liquid–electron crystal” structure based on the mechanism of stabilizing zirconia: (1) The formed Se compound forms a solid solution with β-Cu_2_Se, maintaining the inherent structure in various compositions and temperatures, with no crystal transformation (or very slow transformation). (2) The metal ion radius and Cu^+^ radius are similar, with the difference between them not more than 12%. (3) The Mn–Se bond is more ionizing than that of Cu_2_Se, which has less electronegativity than Cu^+^. Elements in the periodic table with less electronegativity are selected. Then elements with less than a 12% difference between the ionic radius and Cu^+^ are selected from the elements with less electronegativity according to the periodic table of ionic radius. On this basis, through the binary and ternary phase diagrams, the solid solution characteristics with β-Cu_2_Se are determined, and the elements meeting the above three requirements are identified as the candidate elements for stabilizer doping. Mn meets the above three requirements and the relevant parameters of the doping element Mn are shown in Table 1.

The atomic radius of Mn (1.32 Å) is approximately equal to the atomic radius of Cu (1.28 Å), the covalent radius of Mn and Cu are the same, and the ionic radius of Mn^2+^ (0.67 Å) is smaller than that of Cu^+^ (0.77 Å). Therefore, as a dopant, Mn is easily dissolved into the structure of Cu_2_Se. The electronegativity of Mn (1.55 eV) is less than that of Cu (1.90 eV). Theoretically, manganese VIIB has a strong reducing ability, and losing electrons to become manganese ions is more accessible. Mn has a higher valence state (+2, +3, +4, +6, +7) than copper (+1, +2), which is compatible with the idea of doping stable Cu ions with Mn. However, if Mn occupies the Cu position because Mn^2+^ loses more extranuclear electrons than Cu^+^, it increases the electron concentration and thus increases the conductivity. When Mn is doped as a cation, Li Wen [26] et al. showed that Mn doping in SnTe not only adjusted the band structure but also reduced the lattice thermal conductivity, resulting in the alloy form reaching a record *zT*~1.3 at 900 K. Sayan Das [27] et al. investigated the thermoelectric properties of Mn-doped BiCuSeO and found that Mn^2+^ was introduced at the Bi^3+^ site to increase the hole concentration and Seebeck coefficient through heterovalent doping and incorporation of magnetic impurities. The highest power factor of 0.284 mW m^−1^ K^−2^ with *zT* of 0.4 was obtained with Bi_0.92_Mn_0.08_CuSeO at 773 K. Xiang Siqi [28] et al. used Mn doping and S evaporation to improve cuprous sulfide thermoelectric materials’ thermoelectric properties and thermal stability.

In this study, Cu_2−x_Mn_x_Se (x = 0, 0.02, 0.04, 0.06) samples were synthesized by the melting–annealing and SPS methods. With the increase in Mn, the formation of the β-Cu2Se phase is promoted, the diffraction peak shifts to a large angle, and the grain size decreases. The resulting PF value of Cu1.94Mn0.06Se at 873 K was 1.62 mW m^−1^ K^−2^, which is 1.2 times higher than that of Cu_2_Se without Mn at 873 K. Because Mn doping introduces electrons, its bipolar diffusion thermal conductivity significantly increases, resulting in no low thermal conductivity, so Mn-doped samples do not achieve a high *zT* value. According to the results of four cycles, the doped Mn samples show excellent thermal and chemical stability.

## 2. Experiment

### 2.1. Fabrication

**Figure 1:**
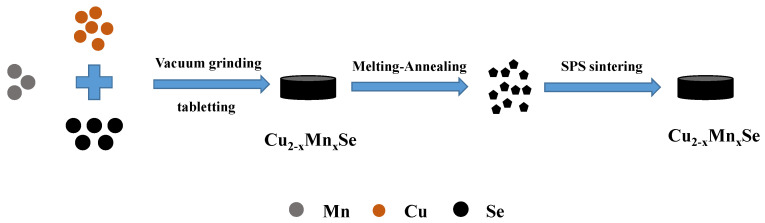
Schematic diagram of the manufacturing process of Mn-doped Cu_2_Se sample.

Polycrystalline samples with nominal compositions of Cu_2−x_Mn_x_Se (x = 0, 0.02, 0.04 and 0.06) were fabricated by a vacuum melting–annealing method and SPS technology. The starting materials (Cu (powder, 99.99%), Se (powder, 99.99%), Mn (powder, 99.99%)) were weighed as per the nominal composition Cu_2−x_Mn_x_Se, thoroughly ground, mixed and cold-pressed in an argon glove box, then loaded into graphite crucibles that were sealed in a fused silica tube under vacuum. The sealed glass tube was placed in the melting furnace, heated to 470 °C at the speed of 10 °C/min, held at 470 °C for 10 h, then heated to 1200 °C at the speed of 10 °C/min, held and melted at 1200 °C for 24 h, and then cooled to 600 °C at the speed of 1 °C/min for 5 days, and finally cooled to room temperature with the furnace. The frit of the desired compound was obtained by cracking the glass tube. After grinding the molten sample into powder, the graphite mold with an inner diameter of 20 mm was sintered in the discharge plasma sintering furnace, and a dense cylindrical block material was obtained. The process parameters of the sintering process are as follows: the average heating rate was 40 °C/min, the sintering temperature was 620 °C, the sintering pressure was 45 MPa, and the heat preservation was 10 min (see Figure 1).

### 2.2. Testing and Characterization

The phase structures of the samples were determined by X-ray diffraction (XRD, Rigaku/SmartLab 3 KW diffractometer Cu-Kα). In order to obtain the cell parameters and other crystal structure information of Cu_2_Se, Rietveld structure refinement was performed on the XRD data for the samples sintered at different temperatures. The microstructure was studied by field emission scanning electron microscopy (FESEM, ZEISS MERLIN Compact, Jena, Germany) and high-resolution transmission electron microscopy (Magellan-400, HRTEM, Titan Themis Z, Thermo Fisher Scientific, Waltham, MA, USA). X-ray photoelectron spectroscopy (XPS, Escalab 250Xi, Thermo Fisher Scientific, USA) was used for analysis of the chemical binding states of the elements. The Seebeck coefficient and electrical conductivity from 303 K to 873 K were measured on a thermoelectric measurement system using (ZEM-3, Ulvac-Riko, Yokohama, Japan). The total thermal conductivity (*κ*) of all the samples was calculated according to the relationship *κ* = *DC_p_ρ*, where *D* is the thermal diffusivity measured by the laser flash analysis (LFA) method (LFA 467; Netzsch, Selb, Germany), *Cp* is the specific heat capacity measured by the LFA method, where a pellet of graphite was used as a reference for *Cp* determination, and *ρ* is the sample density determined using the Archimedes method. The Hall coefficient (*R_H_*) at 303 K and 473 K was measured using a physical properties measurement system (PPMS-9, Quantum Design, San Diego, CA, USA) with a magnetic field from −3 to 3 T. The Hall carrier concentration (*n_H_*) was calculated using *n_H_* = 1/(*R_H_e*), where *e* is the elementary charge. Hall carrier mobility (*μ_H_*) was calculated according to the relation *μ_H_* = *R_H_σ*. The phase transition process was determined by differential scanning calorimeter (DSC, Netzsch, Selb, Germany) under the following conditions: in a nitrogen atmosphere, the rate of temperature rise was 10 °C/min, and the temperature rose from room temperature to 500 °C.

## 3. Results and Discussion

### 3.1. Crystal Structure Analysis

**Figure 2:**
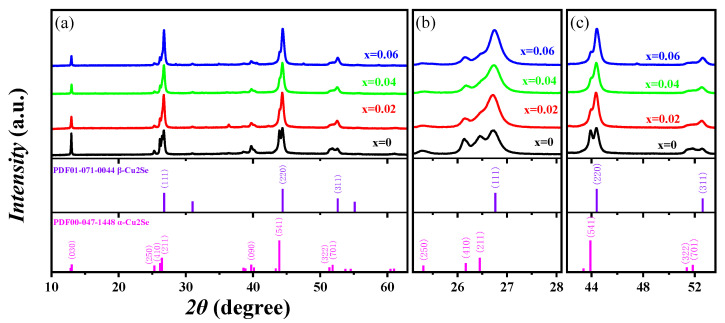
(**a**) Room temperature X-ray diffraction patterns for Cu_2−x_Mn_x_Se. (**b**) Enlarged views of diffraction peaks around 24–28°. (**c**) Enlarged views of diffraction peaks around 43–53°.

**Figure 3:**
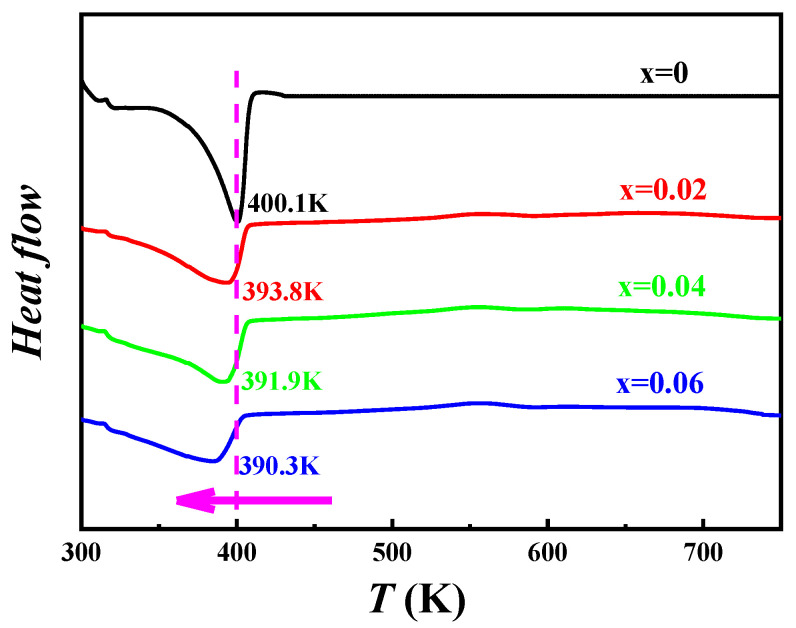
DSC curve and obtained phase transition temperature of Cu_2−x_Mn_x_Se sample.

**Figure 4:**
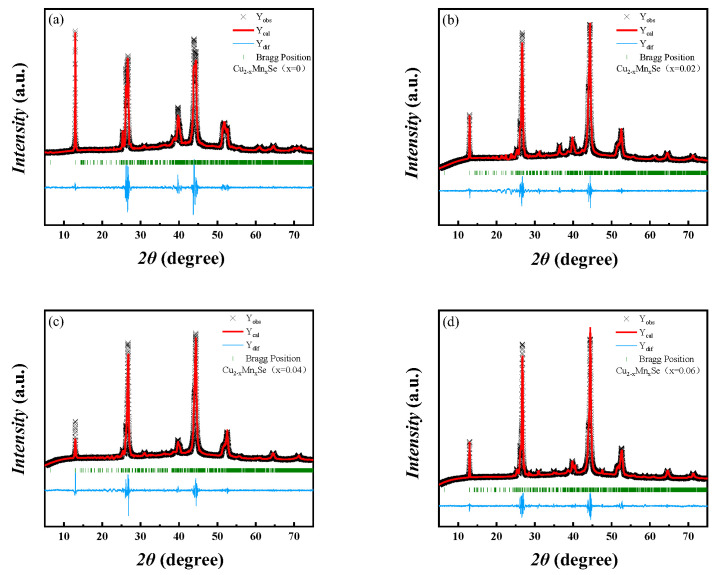
Rietveld refinements of XRD profiles of the Cu_2−x_Mn_x_Se samples. (**a**) x = 0; (**b**) x = 0.02; (**c**) x = 0.04; (**d**) x = 0.06.

Figure 2a shows the XRD pattern of Cu_2−x_Mn_x_Se (x = 0, 0.02, 0.04, 0.06) measured at room temperature. The XRD diffraction peaks coincide with α-phase monoclinic Cu_2_Se (PDF#47-1448) (a = 13.807, b = 20.393, c = 3.923 A) and β-phase Cu_2_Se (PDF#71-0044) (a = b = c = 5.76 A). The diffraction peak intensities of α-Cu_2_Se phase (250) and (211) gradually decrease with the increase in Mn content, while the diffraction peak intensity of β-Cu_2_Se phase (111) gradually increases in Figure 2b. The diffraction peak intensity of the α-Cu_2_Se phase (541) gradually decreases, while that of the β-Cu_2_Se phase (220) gradually increases in Figure 2c. The diffraction peak intensity of the α-Cu_2_Se phase (701) is significantly weakened, enhancing the diffraction peak intensity of the β-Cu_2_Se phase (311). The increase in Mn content induces the advancement of the phase transition from α-Cu_2_Se to the β-Cu_2_Se phase structure, consistent with other reports in the literature [21]. The composition and phase structure of the synthesized sample can be well controlled through Mn blending and vacuum melting–annealing combined with SPS sintering. The β-Cu_2_Se phase can be stabilized with increased Mn. With an increase in Mn, the 2θ angles of the (111), (220), and (311) crystal faces of Cu_2−x_Mn_x_Se are prominent at 26.74°, 44.38°, 52.53°, respectively, and the diffraction peak is shifted to the right or at a large angle. According to the Bragg diffraction equation 2dsinθ = nλ, the cell parameters become smaller because the ionic radii of Cu^+^ and Mn^2+^ are 0.77 Å and 0.67 Å, respectively, the ionic radius of Mn^2+^ is smaller than that of Cu^+^. As the lattice of solid solution doped Mn decreases, the crystal plane spacing decreases, the diffraction angle 2θ increases, and the diffraction angle shifts to a large angle. The (220) and (311) plane diffraction peaks slightly shift in the direction of large angles compared with the standard card. Figure 3 shows the differential scanning calorimetry (DSC) map of Cu_2−x_Mn_x_Se (x = 0, 0.02, 0.04, 0.06). With the increase in Mn doping concentration, the heat absorption peak moves to a lower temperature, and the phase transition temperature decreases from 400.1 K to 390.3 K. It was also shown that Mn doping directly affects the phase transition characteristics. As Mn doping concentration increases, the phase transition temperature decreases, thereby changing from α-phase Cu_2_Se to β-phase Cu_2_Se in advance, which is consistent with the results of XRD. Further, all the XRD patterns were refined and calculated based on the Rietveld refinement. The refinement results are shown in Figure 4, and all samples’ lattice constants and cell volumes were obtained, as shown in Table 2. The refinement results also confirm that compared with the pure Cu_2_Se sample without Mn, the cell volume decreased with the increase in Mn doping content.

**Figure 5:**
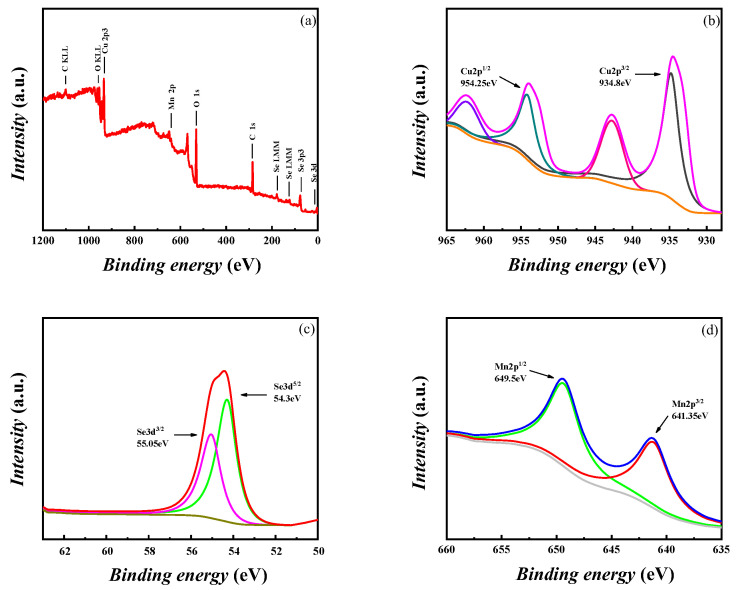
(**a**) XPS spectra of Cu_1.94_Mn_0.06_Se sample, and high-resolution XPS spectra of (**b**) Cu, (**c**) Se and (**d**) Mn.

Figure 5a shows the XPS measurement spectrum of the Cu_1.94_Mn_0.06_Se sample, which proves the existence of the Cu 2p, Se 3d, and Mn 2p energy states. As shown in the Cu 2p XPS spectrum in Figure 5b, the values of the peaks are 934.80 eV and 954.25 eV, which correspond to the respective binding energies of Cu 2p^1/2^ and Cu 2p^1/2^ of the Cu_2_Se lattice in the NIST XPS database, indicating the presence of Cu^+^. As shown in the Se 3d XPS spectrum in Figure 5c, this peak decomposes into two peaks of 54.3 eV and 55.05 eV, corresponding to the binding energies of Se 3d^5/2^ and 3d^3/2^ in the Cu_2_Se lattice, respectively, indicating that Se is in the −2 valence state. The peaks in Figure 5d are 641.35 eV and 649.5 eV, corresponding to the respective binding energies of Mn2p^1/2^ and 2p^3/2^ of the compound. Mn2p^1/2^ indicates the presence of Mn^2+^ ions, which Mn successfully dopes into the Cu_2_Se samples.

### 3.2. Micromorphology Characterization

**Figure 6:**
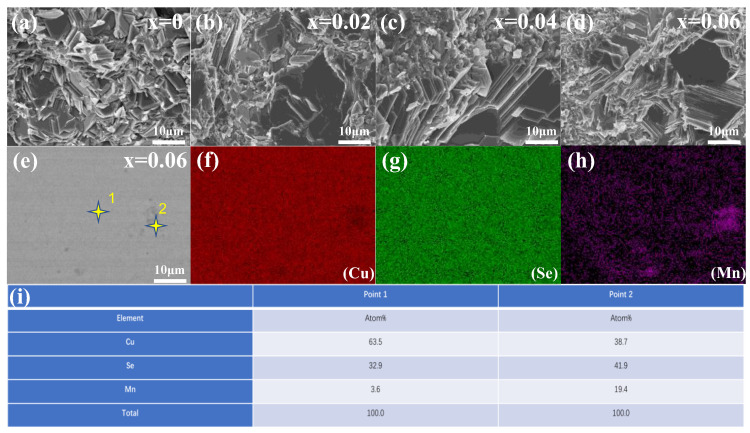
(**a**–**d**) SEM images of Cu_2−x_Mn_x_Se (x = 0, 0.02, 0.04, 0.06) sample fractures; (**e**–**h**) SEM images of the polished surface morphology of Cu_1.94_Mn_0.06_Se samples and the distribution of energy dispersive X-ray spectroscopy (EDS); (**i**) point 1, point 2 element content.

Figure 6 is the SEM diagram of the fracture of the sample. The sintered sample has a typical layered crystal structure, showing the characteristics of layer-by-layer stacking and grains with a fixed orientation, with apparent boundaries, and the appearance of all the samples is the same. In addition to the large flake crystals, there are many small particle wafers, and the size of these small particle wafers decreases with the increase in Mn content. Mn doping increases the melting point of Cu_2_Se, the degree of supercooling increases during the cooling process at the same rate, and the number of nucleations of Cu_2_Se per unit time increases. At the same time, due to the effect of the grain boundary, the difficulty of element diffusion or agglomeration growth increases, so the particle size reduces. Figure 6e–h show the SEM microscopic images of the polished Cu_2−x_Mn_x_Se (x = 0.06) block samples and the corresponding EDS diagrams of Cu, Se, and Mn elements. Irregular dark precipitates ranging in size from submicron to micron are distributed in the Cu_2_Se matrix in Figure 6e. The concentrations of Mn in the light and dark regions of the EDS diagrams in Figure 6f–h indicates an uneven distribution of elements. The EDS data of the gray area (point 1) in Figure 6i shows a Cu-rich phase. The EDS data of the dark region (point 2) in Figure 6i shows a Mn-rich phase.

**Figure 7:**
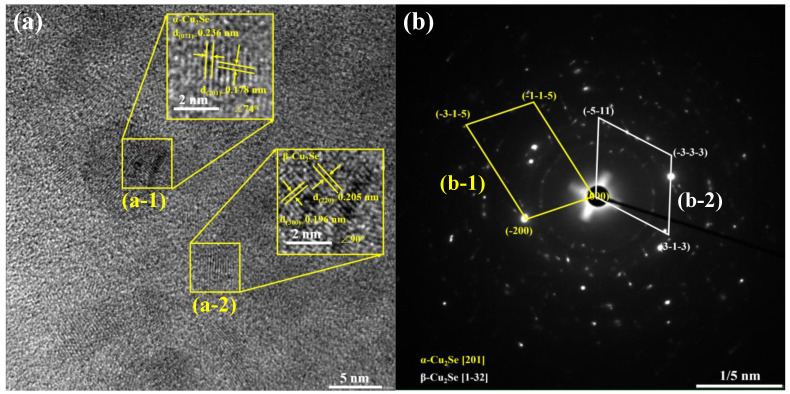
(**a**) HRTEM image of Cu_1.94_Mn_0.06_Se sample, (**b**) SAED images of Cu_1.94_Mn_0.06_Se sample.

We obtained further high-resolution transmission electron microscopy (HRTEM) images of Cu_2−x_Mn_x_Se (x = 0.06). The calculated lattice spacing is comparable to the theoretical crystal plane spacing in Figure 7a, which indicates that (a-1) is the (701) crystal face of α-Cu_2_Se (00-047-1448). The (220) crystal face of β-Cu_2_Se (01-071-0044) in (a-2) is consistent with the XRD results. Selected electron diffraction (SAED) images of the Cu1.94Mn0.06Se sample are shown in Figure 7b. The diffraction point in Figure 7(b-1) well matches the [201] region axis of the α-phase monoclinic Cu_2_Se phase, and the diffraction point in Figure 7(b-2) well matches with the [1-32] region axis of the β-phase cubic Cu_2_Se phase. In the region indicated by a red circle in Figure 7a, the color difference of different micro-regions is likely caused by component segregation. The region has an irregular shape and a size of 5–10 nm. The lattice dislocations and fringes marked by yellow boxes could effectively scatter phonons and reduce lattice thermal conductivity.

### 3.3. Thermoelectric Performance Discussion

**Figure 8:**
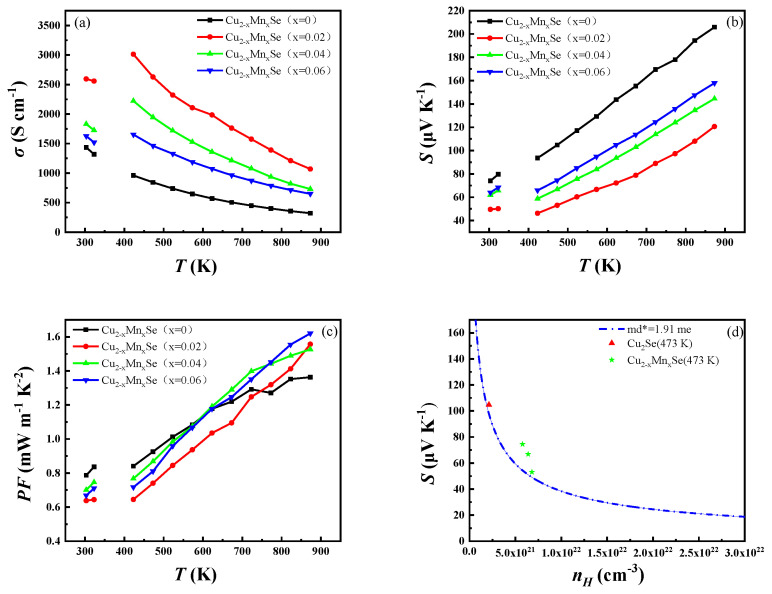
(**a**) Electrical conductivities. (**b**) Seebeck coefficients. (**c**) Power factors. (**d**) Calculated Pisarenko lines of Cu_2−x_MnxSe (x = 0, 0.02, 0.04, 0.06).

The compound’s electrical properties and temperature changes are shown in Figure 6a–d. The TE properties of β-Cu_2_Se after the phase transition are analyzed in detail because of the discontinuity of the phase transition around 400 K. The electrical conductivity of the doped sample, like that of the matrix, gradually decreases with the increase in temperature, showing typical degenerate semiconductor characteristics in Figure 8a. Meanwhile, the electrical conductivity of the doped sample is higher than that of the pure phase Cu_2_Se within the measured temperature range, increasing first with the increase in the impurity content, reaching the maximum value at x = 0.02 and then decreasing. For example, the conductivity at 473 K increases from ~843.1 μV K^−1^ for the x = 0 sample to ~2627.8 μV K^−1^ for the x = 0.02 sample, ~1945.5 μV K^−1^ for the x = 0.04 sample, and ~1461.5 μV K^−1^ for the x = 0.06 sample. Doped Mn occupies the Cu position, Mn^2+^ loses more extranuclear electrons than Cu^+^, or Mn has more extranuclear electrons than Cu, destroying the original charge balance. Each doped or dissolved Mn atom gains one electron, and Mn doping increases the carrier concentration of Cu_2_Se, thereby increasing its conductivity. With the increase in Mn doping, the relative increase in β-Cu_2_Se decreases the carrier concentration and the conductivity of Cu_2_Se. As shown in Figure 8b, the Seebeck coefficients of all the samples are positive throughout the test temperature range, indicating that the holes were the leading carriers. The Seebeck coefficient is opposite to the conductivity, and the Seebeck coefficient of the sample shows a temperature and doping concentration dependence opposite to the conductivity. The Seebeck coefficient increases with the increase in test temperature. With the increase in doping concentration, the Seebeck coefficient of the sample first decreases, reaches the minimum value at x = 0.02 and then increases. The Seebeck coefficient of all doped samples is smaller than that of the pure samples. For example, the Seebeck coefficient decreases from ~104.8 s cm^−1^ for the x = 0 sample to ~53.1 s cm^−1^ for the x = 0.02 sample at 473 K and then increases to ~66.8 s cm^−1^ for the x = 0.04 sample and ~74.5 s cm^−1^ for the x = 0.06 sample.

The power factor values in Figure 8c are calculated according to the formula *PF* = *S*^2^*σ*. The room temperature *PF* of the Cu_2−x_Mn_x_Se solid solution ranges from 0.63 to 0.74 mW m^−1^ K^−2^, which is significantly lower than that of Cu_2_Se. However, the PF significantly improves with the increase in temperature. The solid solution doped with the Mn element is higher than the Cu_2_Se sample at the same temperature at 773 K. The significant increase in σ compensating is attributed to decreased S and improved PF at higher doping levels. Finally, PF reaches the highest value of 1.62 mW m^−1^ K^−2^ for Cu_1.94_Mn_0.06_Se. As shown in Figure 8d, the Pisarenko line (dependent on the S and carrier concentration) of pure phase Cu_2_Se (blue line) with a temperature of 473 K is calculated based on the SPB model. The calculated results are in good agreement with the measured carrier concentration. In contrast, Mn-doped sample (solid green five-pointed star points) is above the Pisarenko line and close to the pure phase curve. The single band model explains the thermoelectric properties of Cu_2_Se doped with Mn. After the phase transition (473 K), the effective mass m_d_^*^ values of the samples doped with x = 0, x = 0.02, x = 0.04, and x = 0.06 Mn are 1.91, 2.32, 2.79 and 2.82*m_e_*, respectively. The effective mass increases with the increase in doping concentration.

(1) $$S=\frac{k_{B}}{e}[\frac{\left(r+\frac{5}{2}\right)F_{\left(r+3/2\right)}\left(\eta \right)}{\left(r+\frac{3}{2}\right)F_{\left(r+1/2\right)}\left(\eta \right)}-\eta ]$$

(2) $$n=\frac{{\left({2m}^{*}k_{B}T\right)}^{3/2}}{3\pi^{2}\hbar^{3}}\frac{{\left(r+3/2\right)}^{2}{F_{\left(r+1/2\right)}}^{2}\left(\eta \right)}{\left(2r+3/2\right)F_{\left(2R+=1/2\right)}\left(\eta \right)}$$

(3) $$F_{j}\left(\eta \right)=\int_{0}^{\infty }\frac{\xi^{j}d\xi }{1+Exp(\xi -\eta )}$$

where *k_B_* is the Boltzmann constant, *e* is the electronic charge, *ħ* is the reduced Planck constant, *η* is the reduced Fermi level, m_d_^*^ is the density of a state’s effective mass, and *F_j_*(*η*) is the *j*th Fermi integral.

**Figure 9:**
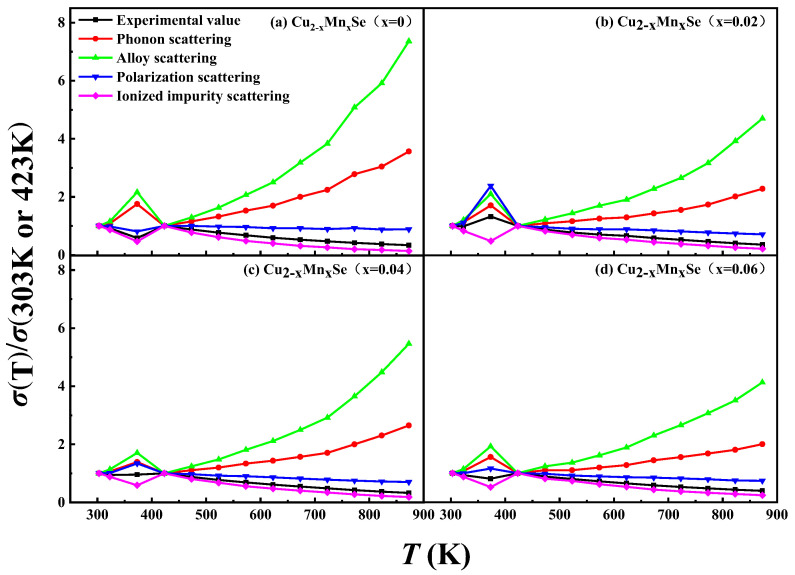
The ratio of the experimental electrical conductivity *σ*(T) to the electrical conductivity at 303 K and 423K as a function of temperature T (line 1) and the calculated ratio values versus T based on acoustic phonon scattering (line 2), alloy scattering (line 3), polar optical phonon scattering (line 4), and ionized impurity scattering (line 5) models for (**a**) Cu_2_Se, (**b**) Cu_1.98_Mn_0.02_Se, (**c**) Cu_1.96_Mn_0.04_Se, (**d**) Cu_1.94_Mn_0.06_Se.

Based on the single band model, the carrier scattering mechanisms of the samples were analyzed by the electrical conductivity ratio method [29], according to Equations (4)–(7) in Table 3, and the results are shown in Figure 9. The electrical conductivity ratios (*σ*(T)/*σ*(T0) of ionized impurity scattering in the test temperature range (303–873 K) for all samples were closest to the experimental values. Therefore, ionized impurity scattering was predominant, and the scattering factor (r) was 3/2. This is one of the reasons why Cu_2_Se had a high Seebeck coefficient.

**Figure 10:**
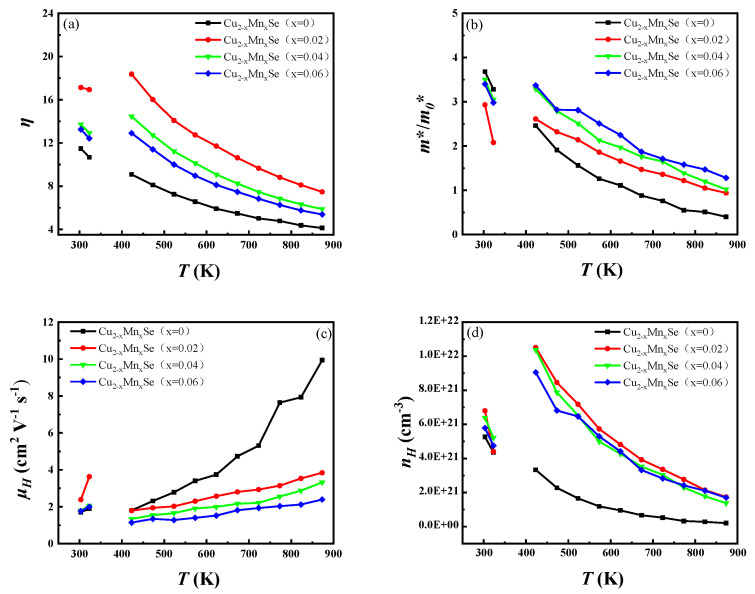
(**a**) Temperature dependences of reduced Fermi level; (**b**) effective mass of Cu_2−x_Mn_x_Se; (**c**) mobility; (**d**) carrier concentration.

To analyze the mechanism of the temperature dependency of electrical conductivity and the Seebeck coefficient, the temperature dependences of the reduced Fermi level (*η = E_F_/k_B_T*), carrier concentration (*n*), carrier mobility (*μ*), and effective mass (m*/m_0_*) of the samples were calculated according to Equations (8)–(12), based on the test results of the electrical conductivities, Seebeck coefficients, and carrier concentrations. The results are illustrated in Figure 10.

(8) $$E_{F}=\pm \frac{\pi^{2}}{3}\frac{{k_{B}}^{2}T(r+\frac{3}{2})}{eS\pm }$$

(9) $${\left(m^{*}\right)}^{1/2}=\frac{3\sigma h^{3}}{e^{2}16\sqrt{2}\pi \tau_{0}{E_{F}}^{r+\frac{3}{2}}}$$

(10) $$\tau_{0}=\frac{3\sigma h^{3}}{16\pi \sqrt{2}e^{2}{\left(m^{*}\right)}^{1/2}{E_{F}}^{r+\frac{3}{2}}}$$

(11) $$n=\frac{\pi }{3}\frac{{\left(8m^{*}E_{F}\right)}^{3/2}}{h^{3}}$$

(12) $$\mu =e{\left(m^{*}\right)}^{-1}\tau_{0}{E_{F}}^{s}$$

where *σ* is the conductivity, *k_B_* is the Boltzmann constant, *T* is the temperature in Kelvin, *e* is the electron charge, *τ*_0_ is the constant, m* (DOS) is the effective mass, *E_F_* is the Fermi level, *r* is the scattering factor, and *h* is Planck’s constant.

It can be seen in Figure 10a that each sample’s reduced Fermi level is much higher than 3. Therefore, it is reasonable to select the degenerate calculation model. The reduced Fermi level decreases with the increase in test temperature, indicating that the degree of degeneracy decreases. However, it increases with Mn doping, indicating that it will increase its degeneracy and carrier concentration. The effective mass of the state density of the carrier presents the complete opposite change rule to that of mobility in Figure 10b. It decreases with the increase in temperature and increases with the increase in Mn doping amount. According to the single parabolic band model, under the scattering condition of ionized impurities, the effective mass of the state density m^*^, conductivity and Fermi level follow the relationship shown in Equation (13):

(13) $$({m^{*})}^{\frac{1}{2}}=\frac{3\sigma h^{3}}{16\sqrt{2}\pi e^{2}\tau_{0}E_{F}^{3}}$$

m^*^ is directly proportional to the conductivity and inversely proportional to the Fermi level. According to Figure 10a, the conductivity of each sample decreases rapidly with the increase in temperature, while its *E_F_* increases slightly with the increase in temperature. This shows that Mn doping can improve the effective mass of carrier state density, which is conducive to increasing the Seebeck coefficient of Cu_2_Se. However, as mentioned before, Mn doping causes an increase in the carrier concentration, especially the concentration of a few carriers, so the amount of Mn doping is crucial. It can be seen in Figure 10c that the carrier mobility increases with the increase in temperature, which is reflected in the scattering of ionized impurities. This result is consistent with the carrier scattering mechanism analysis results based on the conductivity ratio method mentioned above. With the increase in Mn concentration, the mobility of the sample decreases. On the one hand, the alloy scattering increases. On the other hand, the grain boundary and impurity scattering to the carrier increase. As shown in Figure 10d, the carrier concentration decreases with the increase in test temperature, which is mainly caused by the decrease in Cu^+^ vacancies with the increase in temperature, and a defect reaction occurs, as shown in Equation (14):

(14) $$Cu_{surface}+V_{Cu}^{\prime }+h=Cu_{Cu}$$

The carrier concentration of the doped sample is higher than that of the pure sample in the whole test temperature range. With the increase in doping, the carrier concentration first increases, reaches the maximum at x = 0.02 and then decreases. Because the ionic radius of Mn^2+^ is smaller than that of Cu^+^, doping Mn can occupy not only the Cu vacancy but also the lattice site of Cu, resulting in defect reaction Equations (15) and (16), respectively. Mn^2+^ loses more extranuclear electrons than Cu^+^, or Mn has more extranuclear electrons than Cu, destroying the original charge balance. The result is that each Mn atom doped or dissolved produces one electron. Equation (15) does not reduce the carrier concentration, and Equation (16) increases the carrier concentration, so Mn doping increases the carrier concentration of Cu_2_Se, and thus increases its conductivity.

Mn + V’_Cu+_ + h →Mn˙_Cu+_ + e (x − y > 0) (15)

xMn + Cu_2−x_Se → xMn˙_Cu+_ + (2 − x)Cu_Cu_ + Se_Se_ + xe (16)

**Figure 11:**
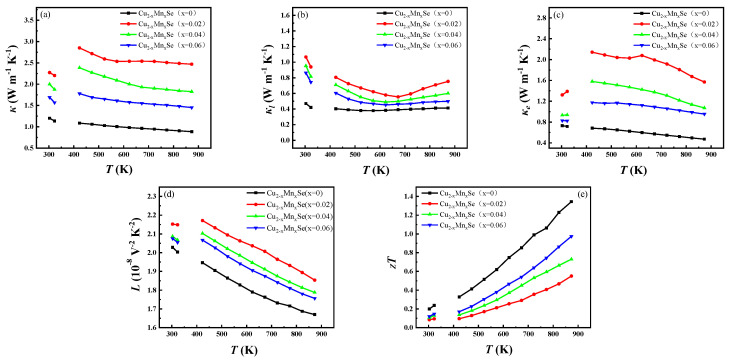
(**a**) Temperature dependence of total thermal conductivity; (**b**) the lattice thermal conductivity; (**c**) the electronic thermal conductivity; (**d**) Lorentz constant; (**e**) *zT* value.

Figure 11a shows the variations in thermal conductivity with the test temperature for the Cu_2−x_Mn_x_Se (x = 0, 0.02, 0.04, 0.06) samples. Compared with pure Cu_2_Se, the total thermal conductivity of the sample containing Mn shows a temperature- and doping concentration-dependent relationship, with its conductivity decreasing with increasing temperature. As the doping concentration increases, it reaches a maximum value at x = 0.02 and then decreases but remains higher than that of the pure sample throughout the measured temperature range. The main reason is that the electrical conductivity of the doped samples is higher than that of the pure Cu_2_Se samples. For example, at 473 K, the total thermal conductivity increases from ~1.06 W m^−1^ K^−1^ for the original Cu_2_Se to ~2.71 W m^−1^ K^−1,^ for the x = 0.02 sample, to ~2.27 W m^−1^ K^−1^ for the x = 0.04 sample, to ~1.69 W m^−1^ K^−1^ for the x = 0.06 sample. Because Mn doping introduces a large number of minority carriers or electrons, its total thermal conductivity (*κ_b_*) mainly consists of lattice thermal conductivity (*κ_l_*), carrier thermal conductivity (*κ_e_*), and bipolar diffusion thermal conductivity *κ_b_*, that is, *κ* = *κ_l_* + *κ_e_* + *κ_b_*. In order to fully understand the change in total thermal conductivity, the κ_e_ and *κ_l_* + *κ_b_* of the sample are analyzed in Figure 11b,c. According to Wiedemann-Franz’s law, *κ_e_* = *LσT*, where L is the Lorentz constant, and the Lorentz constant *L* (10^−8^ W Ω K^−1^) = 1.5 + exp (−*S*/116). The calculation results are shown in Figure 11d. The lattice thermal conductivity and the sum of the bipolar diffusion thermal conductivity (*κ_l_* + *κ_b_*) of the doped samples are higher than those of the undoped Cu_2_Se samples, and the lattice thermal conductivity increases first with the increase in Mn concentration, reaches the maximum value at x = 0.02, and then decreases. The first reason is consistent with the results of the SEM microstructure analysis, that is, with the increase in Mn doping content, the decrease in grain size, the increase in composition heterogeneity, and the increase in lattice defects such as dislocation enhance the lattice scattering of phonons and reduce the lattice thermal conductivity. Second, Mn doping introduces a small number of carriers, the concentration of which first increases with the increase in the x value, reaches a maximum value at x = 0.02, and then decreases with the increase in Mn doping content and Mn solid solubility. The increase in minority carrier concentration increases the bipolar diffusion thermal conductivity, and *κ_l_* + *κ_b_* increases compared with the undoped sample, reaching a maximum value at x = 0.02.

As shown in Figure 11e, the *zT* values of the Mn-doped samples are significantly lower than those of the pure Cu_2_Se samples over the entire temperature range. Mn doping increases the power factor of samples due to the increase in electrical conductivity. However, due to the influence of bipolar diffusion thermal conductivity, Mn doping does not reduce the thermal conductivity, so the ideal *zT* value is not obtained.

### 3.4. Cyclic Test of Thermoelectric Properties

**Figure 12:**
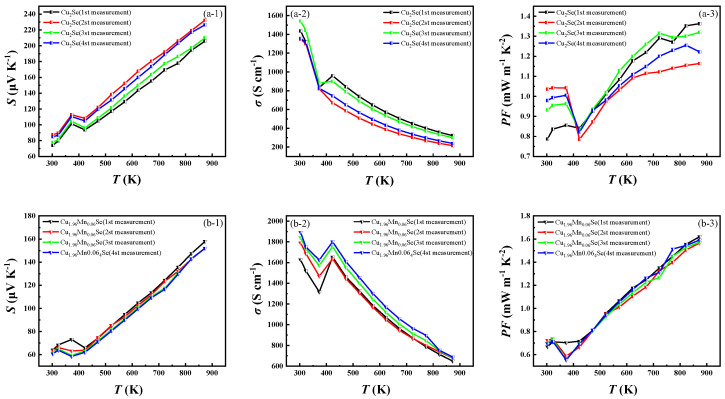
Cyclic tests of Cu_2_Se and Cu_1.94_Mn_0.06_Se. (**a-1**,**b-1**) Seebeck cycle test; (**a-2**,**b-2**) conductivity cycle test; (**a-3**,**b-3**) *PF* calculation results after the cycle.

In order to verify the effect of Mn doping on Cu^+^ migration, the thermoelectric properties of Cu_2_Se and Cu_1.94_Mn_0.06_Se were tested four times and the results are shown in Figure 12. The variation range of *S*, *σ*, and *PF* of doped Cu_1.94_Mn_0.06_Se after multiple cycles is significantly smaller than that of pure Cu_2_Se. The pyroelectric properties of Cu_1.94_Mn_0.06_Se after four cycles of testing show little change. Cu_1.94_Mn_0.06_See has better cyclic stability at high temperatures than Cu_2_Se. Mn doping can effectively reduce Cu^+^ migration and provide the experimental basis for improving its thermoelectric stability.

## 4. Conclusions

By vacuum melting–annealing synthesis and the SPS sintering process, Cu_2−x_Mn_x_Se (x = 0, 0.02, 0.04, 0.06) semiconductors were fabricated successfully. The crystal structure, microstructure, thermoelectric properties, carrier scattering mechanism, and energy band parameters of Mn-doped samples were studied at a range of test temperatures (303–873 K) and doping amounts of Mn. The thermal stability of the Mn-doped samples was studied by cyclic testing of high-temperature thermoelectric properties. Conclusions are drawn as follows:

1. XRD analysis shows that the Mn-doped samples are a mixture of α-Cu_2_Se and Cu_2_Se. With the increase in Mn content, the cell parameters of the material decrease, and the β-Cu_2_Se phase content increases. Specifically, with the increase in Mn content, the β-Cu_2_Se phase can be stabilized theoretically. DSC analysis showed that the heat absorption peak moves to low temperatures, and the phase transition temperature decreases with the increase in Mn doping content.
2. SEM and HRTEM analysis showed that the sample is composed of flaky crystal piles. the flaky particles decreased with the increase in Mn doping amount; the shape of the segregation region of the sample was irregular; the size was 5–10 nm; and there were many dislocations in each segregation micro-interval, which enhanced the scattering of carriers and phonons.
3. The σ and S showed opposite test temperature and Mn doping dependence. The conductivity decreases with the increase in temperature, and S increases with the increase in temperature. The PF value of Cu1.94Mn0.06Se at 873 K was 1.62 mW m^−1^ K^−2^, which is 1.2 times higher than that of Cu2Se without Mn at 873 K.
4. Conductivity ratio analysis showed that the Mn impure sample and pure phase Cu_2_Se have the exact carrier scattering mechanism, the scattering characteristic of ionized impurities. This is consistent with the result that the carrier mobility increases with temperature, so the scattering factor value is 3/2. This feature could be one of the reasons for Cu_2_Se’s high Seebeck coefficient and thermoelectric properties.
5. Due to Mn’s inclusion of electrons, its bipolar diffusion thermal conductivity significantly increases. The minority carrier concentration introduced at x = 0.02 is the highest for the lattice thermal conductivity of the sample, which does not reach a high *zT* value.
6. The results of four high-temperature cycle tests showed that the thermoelectric properties of Cu_1.94_Mn_0.06_Se show little change, and the high-temperature cycle stability is better than that of Cu_2_Se. The ionic radius of Mn^2+^ is smaller than that of Cu^+^. Therefore, as a dopant, Mn is easily dissolved into the structure of Cu_2_Se. The electronegativity of Mn is less than that of Cu. Theoretically speaking, the manganese VIIB element has a strong reducing ability.

## Figures and Tables

**Table 1:** Parameters of doped elements.

Element	Cu	Mn	Cu^+^	Cu^2+^	Mn^2+^
radius/Å [24]	1.28	1.32	0.77	0.73	0.67
electronegativity/eV [25]	1.90	1.55	1.8	2.0	1.4

**Table 2:** Lattice constants of Cu_2−x_Mn_x_Se samples at room temperature.

Sample	a (Å)	b (Å)	c (Å)	Vol (Å^3^)	χ^2^
x = 0	7.159418	12.002411	27.771622	2345.075	4.151
x = 0.02	7.005779	12.408270	27.327600	2344.639	4.165
x = 0.04	6.988073	12.514909	27.393709	2244.077	4.626
x = 0.06	7.373002	12.079905	27.417471	2243.847	4.028

**Table 3:** Formulas used for carrier scattering mechanism analysis.

Carrier Scattering Mechanism	Scattering Factor	Electrical Conductivity
Acoustical phonon scattering	−1/2	$\sigma =\sigma_{0}\frac{\eta }{m^{*}}$ (4)
Alloy scattering	−1/2	$\sigma =\sigma_{0}\frac{T\eta }{m^{*}}$ (5)
Polar optical phonon scattering	1/2	$\sigma =\sigma_{0}T^{2}\eta^{2}$ (6)
Ionized impurity scattering	3/2	$\sigma =\sigma_{0}m^{*}T^{3}\eta^{3}$ (7)

## Data Availability

Not applicable.

## References

[1] Mao J., Chen G., Ren Z. (2021). Thermoelectric cooling materials. Nat. Mater..

[2] Zhao K., Qiu P., Shi X., Chen L. (2020). Recent Advances in Liquid-Like Thermoelectric Materials. Adv. Funct. Mater..

[3] Zhang Z., Zhao K., Wei T.-R., Qiu P., Chen L., Shi X. (2020). Cu_2_Se-Based liquid-like thermoelectric materials: Looking back and stepping forward. Energy Environ. Sci..

[4] Pei Y., Wang H., Snyder G.J. (2012). Band Engineering of Thermoelectric Materials. Adv. Mater..

[5] Lu R., Olvera A., Bailey T.P., Uher C., Poudeu P.F. (2020). Nanoscale Engineering of Polymorphism in Cu_2_Se-Based Composites. ACS Appl. Mater. Interfaces.

[6] Liu H., Shi X., Xu F., Zhang L., Zhang W., Chen L., Li Q., Uher C., Day T., Jeffrey G.S. (2012). Copper ion liquid-like thermoelectrics. Nat. Mater..

[7] Wei T.-R., Wu C.-F., Li F., Li J.-F. (2018). Low-cost and environmentally benign selenides as promising thermoelectric materials. J. Mater..

[8] Bo L., Zhang R., Zhao H., Hou Y., Wang X., Zhu J., Zhao L., Zuo M., Zhao D. (2022). Achieving High Thermoelectric Properties of Cu_2_Se via Lattice Softening and Phonon Scattering Mechanism. ACS Appl. Energy Mater..

[9] Byeon D., Sobota R., Delime-Codrin K., Choi S., Hirata K., Adachi M., Kiyama M., Matsuura T., Yamamoto Y., Matsunami M. (2019). Discovery of colossal Seebeck effect in metallic Cu_2_Se. Nat. Commun..

[10] Zhao K., Blichfeld A.B., Eikeland E., Qiu P., Ren D., Iversen B.B., Shi X., Chen L. (2017). Extremely Low Thermal Conductivity and High Thermoelectric Performance in Liquid-like Cu_2_Se _1−x_S_x_ Polymorph Materials. J. Mater. Chem. A.

[11] Zhang J., Zhang C., Zhu T., Yan Y., Su X., Tang X. (2021). Mechanical Properties and Thermal Stability of the High-Thermoelectric-Performance Cu_2_Se Compound. ACS Appl. Mater. Interfaces.

[12] Peng P., Gong Z.N., Liu F.S., Huang M.J., Ao W.Q., Li Y., Li J.Q. (2016). Structure and thermoelectric performance of β-Cu_2_Se doped with Fe, Ni, Mn, In, Zn or Sm. Intermetallics.

[13] Wang N., Song G., Li G., Wu Y., You J. (2022). Thermoelectric properties of β-(Cu,Mn)_2_Se films with high (111) preferred orientation. Vacuum.

[14] Ducka A., Trawiński B., Bochentyn B., Dubiel A., Kusz B. (2021). Structure and thermoelectric properties of nickel-doped copper selenide synthesised in a hydrogen atmosphere. Mater. Res. Bull..

[15] Day T.W., Borup K.A., Zhang T., Drymiotis F., Brown D.R., Shi X., Chen L., Iversen B.B., Snyder G.J. (2014). High-temperature thermoelectric properties of Cu_1.97_Ag_0.30_Se_1+y_. Mater. Renew. Sustain. Energy.

[16] Hu Q., Zhu Z., Zhang Y., Li X.-J., Song H., Zhang Y. (2018). Remarkably high thermoelectric performance ofCu_2−x_Li_x_Se bulks with nanopores. J. Mater. Chem. A.

[17] Zhu Z., Zhang Y., Song H., Li X.-J. (2019). High thermoelectric performance and low thermal conductivity in Cu_2−x_Na_x_Se bulk materials with micro-pores. Appl. Phys. A.

[18] Zhu Z., Zhang Y., Song H., Li X.-J. (2018). Enhancement of thermoelectric performance of Cu_2_Se by K doping. Appl. Phys. A.

[19] Bhardwaj R., Bhattacharya A., Tyagi K., Gahtori B., Chauhan N.S., Vishwakarma A., Johari K.K., Bathula S., Auluck S., Dhar A. (2019). Enhancement in thermoelectric performance of single step synthesized Mg doped Cu_2_Se: An experimental and theoretical study. Intermetallics.

[20] Wang J., Liu B., Miao N., Zhou J., Sun Z. (2019). I-doped Cu_2_Se nanocrystals for high-performance thermoelectric applications. J. Alloys Compd..

[21] Kim M.J., Lee G.-G., Kim W., Kim K., Tak J.-Y., Shin W.H., Seo W.-S., Hong J., Lim Y.S. (2018). Effects of Cl-Doping on Thermoelectric Transport Properties of Cu_2_Se Prepared by Spark Plasma Sintering. J. Electron. Mater..

[22] Zhao K., Blichfeld A.B., Chen H., Song Q., Zhang T., Zhu C., Ren D., Hanus R., Qiu P., Iversen B.B. (2017). Enhanced Thermoelectric Performance through Tuning Bonding Energy in Cu_2_Se_1−x_S_x_ Liquid-like Materials. Chem. Mater..

[23] Butt S., Xu W., Farooq M.U., Ren G.K., Zhang Q., Zhu Y., Khan S.U., Liu L., Yu M., Mohmed F. (2016). Enhanced Thermoelectricity in High-Temperature β-Phase Copper(I) Selenides Embedded with Cu_2_Te Nanoclusters. ACS Appl. Mater. Interfaces.

[24] James G., Speight P.D. (1972). Lange’s Handbook of Chemistry.

[25] Gordy W., Thomas W.J.O. (1956). Electronegativities of the Elements. J. Chem. Phys..

[26] Li W., Chen Z., Lin S., Chang Y., Ge B., Chen Y., Pei Y. (2015). Band and scattering tuning for high performance thermoelectric Sn_1−x_Mn_x_Te alloys. J. Mater..

[27] Das S., Valiyaveettil S.M., Chen K.-H., Suwas S., Mallik R.C. (2019). Thermoelectric properties of Mn doped BiCuSeO. Mater. Res. Express.

[28] Xiang S., Liang Y., Zhang X. (2022). Thermoelectric properties and thermal stability of metal-doped cuprous sulfide thermoelectrics. J. Eur. Ceram. Soc..

[29] Xu G., Ren P., Lin T., Wu X., Zhang Y., Niu S., Bailey T.P. (2018). Mechanism and application method to analyze the carrier scattering factor by electrical conductivity ratio based on thermoelectric property measurement. J. Appl. Phys..

